# Evaluating the impact of easy-to-understand *patient letters* after discharge on patients’ health literacy: a randomized controlled study

**DOI:** 10.1186/s12913-025-13464-4

**Published:** 2025-10-15

**Authors:** Henna Riemenschneider, Annika Rettich, Henriette Hoffmann, Rebekka Post, Ronny Zenker, Karen Voigt, Antje Bergmann, Ansgar Jonietz

**Affiliations:** 1 Department of General Practice, Faculty of Medicine, University Hospital Carl Gustav Carus, TUD Dresden University of Technology, Fetscherstraße 74, 01307 Dresden, Germany; 2 “Was hab’ ich?” gGmbH, Dresden, Germany

**Keywords:** Discharge letter, Patient letter, Health communication, Health literacy, Discharge management, Patient empowerment

## Abstract

**Background:**

More than half of the German population has considerable difficulties in understanding health information, reflecting limited health literacy, which is associated with poorer health outcomes. Personalized, automatically generated, easy-to-understand discharge letters (*patient letters*) are designed to improve patients’ comprehension of medical information after hospital discharge. This study investigated whether these letters improve health literacy in cardiologic patients and explored their perceptions.

**Methods:**

This randomized controlled study included 738 patients discharged from a heart center in Dresden, Germany. The control group (CG; *n* = 375) received a conventional discharge letter only, whereas the intervention group (IG; *n* = 363) additionally received a software-generated *patient letter* by post. Five to nine days later, participants received a sociodemographic survey and the HLS-EU-Q16 health literacy questionnaire. IG participants additionally evaluated the *patient letter*, and *n* = 15 were interviewed qualitatively to gain deeper insights.

**Results:**

Post-intervention, IG participants had significantly higher health literacy than CG participants (*p* = .002, Mann-Whitney *U* test). Over 90% of IG participants rated the *patient letter* as helpful, comprehensible and informative. Qualitative interviews revealed a largely positive attitude toward *patient letters*, along with some suggestions for content improvement.

**Conclusions:**

*Patient letters* were associated with higher post-intervention health literacy in cardiologic patients and were well received. These results suggest that broad implementation of *patient letters* may benefit both patients and healthcare providers by enhancing patients’ understanding of medical information regarding diagnoses and treatment, potentially contributing to improved health-related outcomes.

**Trial registration:**

The study was retrospectively registered in the German Clinical Trials Register on December 13, 2024 under the number DRKS00035706.

**Supplementary Information:**

The online version contains supplementary material available at 10.1186/s12913-025-13464-4.

## Background

Medical terminology is often difficult for lay people to understand. This can lead to situations where patients struggle to fully comprehend their own diagnoses, examinations or treatments performed during a hospital stay. In the German healthcare system, it is common practice that patients receive a discharge letter at the end of their hospital stay, summarizing all diagnoses and treatments carried out. However, similar to countries like the US, Canada or the UK, these discharge letters are primarily intended for communication among healthcare providers and are commonly not designed in a patient-oriented way [1–4]. They often contain complicated medical language, are generally not uniformly structured and can vary greatly in quality and detail [2]. Emphasizing the urgency of improving health literacy among patients, the 91st Conference of German Health Ministers [5] declared that every patient should receive an easy-to-understand document after inpatient treatment. In line with this, the non-profit company “Was hab’ ich?” (“What’s my diagnosis?”) developed personalized, automatically generated, easy-to-understand discharge letters (short: *patient letters*), which are supplementary patient-friendly documents that simplify conventional discharge information. These letters break down the diagnoses, examinations and treatments carried out in an easy-to-understand language. In order to implement such an intervention in regular clinical care, it must be feasible in daily practice. Accordingly, the “Was hab’ ich?” *patient letters* were developed in such a way that their implementation virtually requires no additional effort on part of the medical staff. The simplified wording simplified wording used in *patient letters* is supposed to enable patients to gain a better understanding of personal medical information and result in an overall enhancement of their individual level of health literacy.

### Concept of health literacy

Health literacy includes the knowledge, motivation and ability to find, understand, evaluate and apply health-related information [6]. It is a multidimensional concept, as it takes into account personal resources but also contextual factors. This means that health literacy can be understood as an interplay of a person’s skills or abilities (personal health literacy) and the demands placed on an individual in the respective systems, organizations and living environments in which they reside (systemic/organizational health literacy; [7, 8]). Following this definition, health literacy can be increased via two different mechanisms: first, by training personal skills and abilities, second, by creating healthcare environments that are adequately adapted to different skills and abilities.

### Lack of health literacy in general population

Past research has consistently shown a substantial lack of health literacy in the general population across many countries [9–12]. More than half of German adults report considerable difficulties in dealing with health-related information [13]. Low health literacy also has significant consequences in hospital settings on an international scale. After discharge, many patients exhibit deficits in comprehending medication instructions [14], acknowledging home care requirements and recognizing when clinical re-presentation is warranted [15]. A lack of health literacy was shown to be associated with poorer health-related outcomes and higher overall costs for the healthcare system [10–13]. Hence, it is in the interest of both patients and healthcare providers to increase the level of health literacy in the general population. Strengthening health literacy was consequently declared an urgently needed health policy goal by the “Nationaler Aktionsplan Gesundheitskompetenz für Deutschland” (*National Action Plan on Health Literacy for Germany*) in 2018. One of the 15 recommendations made in this context addresses the need to make health information more user-friendly so that patients can better understand, assess and use information. This includes the use of easy-to-understand language as well as providing all health-related information in written form for patients [13, 16]. The implementation of patient-friendly discharge letters could contribute to this aimed-for improvement in health literacy through two mechanisms: first, by enhancing personal diagnosis- and treatment-related knowledge, and second, by reducing barriers to understanding medical information through a simplified wording of medical terminology. In this way, such letters can contribute to both personal and organizational health literacy by improving how medical information is communicated.

### Previous studies on patient-friendly discharge letters

In the past, there have been individual efforts to develop and test patient-friendly discharge documents to be issued to patients after discharge from hospital. One study carried out at the University Medical Center Hamburg-Eppendorf, Germany [17] found that a patient-friendly discharge letter combined with a systematic discharge discussion improved patient compliance with behavioral recommendations. Another study conducted at Großhadern Hospital, Germany [18] showed that patients who received patient-friendly discharge letters were significantly more satisfied with the information provided and had a higher level of knowledge about their diagnoses compared to those who received standard discharge letters. Patient-friendly discharge letters in this study contained a brief summary of patients’ diagnoses and selected information considered relevant for their recovery. A third study – a previous project by “Was hab’ ich?” [19] – investigated the effect of a personalized, easy-to-understand *patient letter*, similar to the ones used in the present study, but in an earlier version that was assembled manually. While the results did not show an overall improvement in self-reported health literacy (24 selected items of the HLS-EU-Q47), positive effects were observed for individual items, such as comprehension of medical advice and understanding and implementing medical instructions. Additionally, over 95% of patients rated the letters positively, finding them informative, understandable, and helpful. International research further supports these findings, with a review from 2019 showing that patients generally rate patient-friendly discharge letters positively [20]. The authors further emphasized their potential to work as a reference and reminder medium. An Australian study in 2014 found that patient-friendly discharge letters enhance understanding of illness and treatment [21]. A 2006 study explored the impact of patient-oriented summaries of consultations, finding that while not exhibiting a measurably effect on recall, patients expressed a high satisfaction and found the summary letters useful for understanding and sharing medical information [22]. In a qualitative study from Denmark in 2021, patients receiving personalized discharge letters reported feeling more secure and better able to communicate with healthcare providers [23].

It is crucial to note that in all of these studies, patient-friendly discharge letters served as a supplement to (and not a replacement for) conventional discharge letters and were created in a manual process, requiring significant additional effort. This could explain why, despite the shown beneficial effects, such interventions have not been widely implemented outside of research settings.

### Research question: which benefits can patient letters provide?

Based on the promising findings of the previous studies on patient-friendly discharge letters, we assumed that the personalized, automatically generated *patient letters* by “Was hab’ ich?” can have positive effects on health communication and various clinically relevant factors, such as patient compliance, personal satisfaction as well as specific subscales of health literacy. Yet, to date, no study could show any patient-friendly discharge letters to lead to an improvement in overall health literacy. With the current study, we aimed to investigate whether the easy-to-understand approach (simplified wording of detailed, individualized medical information) of the *patient letters* would lead to an increase in patients’ self-assessed understanding of medical and health-related information and consequently would have a positive effect on self-reported health literacy. In this light, we first hypothesized that receiving a *patient letter* after an inpatient stay would increase self-reported health literacy in patients. Second, we hypothesized that patients would perceive the received *patient letter* as understandable, helpful and informative.

## Methods

### Study design

**Fig. 1:**
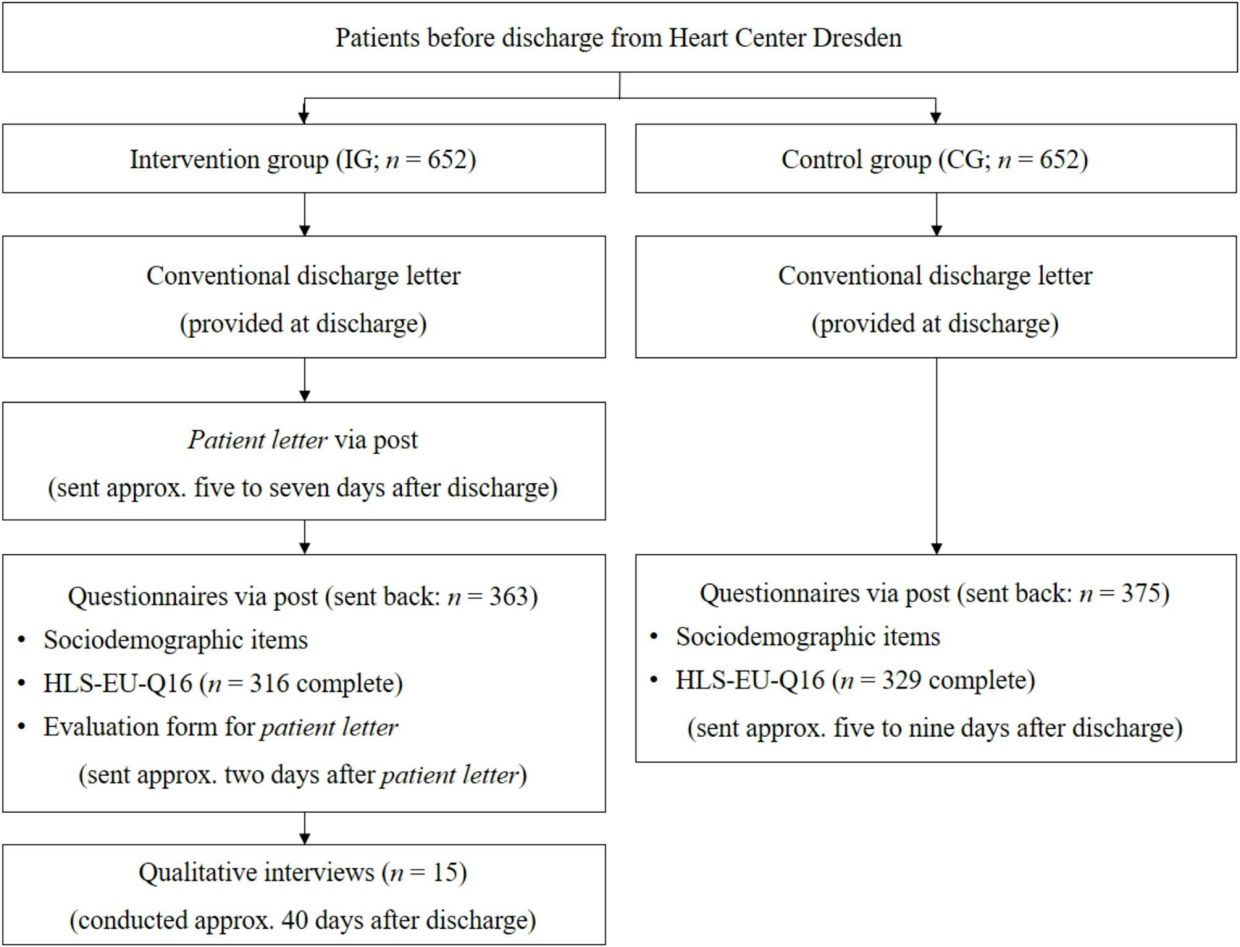
Course of the study

From June 2019 to June 2020, a randomized controlled study was conducted at the Heart Center Dresden, Germany, using a mixed-methods approach with an explanatory sequential design. Participating patients were equally assigned to either intervention group (IG) or control group (CG) using computer-generated block randomization with blocks of 20 to ensure equal group sizes while maintaining random allocation. Following standard clinical practice, both groups received a conventional discharge letter after their inpatient stay. IG participants additionally received a personalized, automatically generated, easy-to-understand discharge letter (*patient letter*) that was sent around five to seven days after discharge by post. Approximately five to nine days after discharge, both groups were sent a selection of questionnaires (see Materials and instruments). Additionally, participants of IG were invited to qualitative guided interviews conducted by trained project staff in order to gain more profound insights into their attitudes towards *patient letters* and identify potential needs for improvement. Participants did not receive any financial incentives. All quantitative and qualitative data were pseudonymized using a unique code following data protection standards to ensure the confidentiality of sensitive patient information. The course of the study is displayed in Fig. 1.

### Sample description

The required sample size of at least *n* = 357 participants per group was calculated using the G*Power software [24]. The estimated effect size of Cohen’s *d* = 0.21 for a group difference in the HLS-EU-Q16 overall score was retrieved from the effect found in the “Was hab’ ich?” pilot study [19]. The subset of participants who sent back their questionnaires consisted of *n* = 363 in the IG and *n* = 375 in the CG. For the qualitative interviews, IG participants were invited, whereby a heterogeneous distribution in terms of age, gender and level of education was ensured in order to achieve a potentially high degree of variability in the responses. The number of interviews emerged during the process: interviews were conducted until theoretical saturation was reached. Qualitative analysis took place parallel to the interview period in order to be able to make prompt decisions on the theoretical saturation.

We recruited participants among planned inpatients with cardiovascular diseases, which were regularly admitted and discharged at the Heart Center Dresden between the beginning of June 2019 and the end of June 2020. As recruitment coincided with the early months of the COVID-19 pandemic, it is possible that the pandemic influenced patient admissions and selection. However, since the study was conducted in a specialized healthcare institution where the impact on daily procedures was lower than in other healthcare settings (e.g., primary care), we consider the impact to be negligible. Inclusion criteria were a minimum age of 18 years and sufficient German language skills to understand the study documents without an interpreter. Further, participants had to give a written informed consent to participate in the study which included an acknowledgment of their understanding of the study’s purpose, procedures, use of data for scientific purposes, and their right to withdraw from the study at any time without consequences. Exclusion criteria were admission through emergency, return of the questionnaire after the specified time frame (> 40 days post-discharge) and withdrawal or lack of consent to participate in the study.

### Materials and instruments

The software used to generate automated *patient letters* was developed by the non-profit company “Was hab’ ich?” [25]. The software uses discharge data, including standardized codes for diagnoses, operations and procedures that are documented for all patients in German hospitals for billing purposes. Yet, the letters do not merely provide a lay-friendly summary of the conventional discharge letter, but also contain additional information boxes with individually relevant disease-specific background information. For the purpose of this study, the software was locally connected to the hospital information system. Immediately after completion of the final administrative documentation, the documented codes could be authorized to be linked to predefined text modules through the software. Using rule-based algorithms, text modules were adapted based on each patient’s specific combination of codes and compiled into a personalized *patient letter*. The letters were printed out directly in the clinic, ensuring that patient data remained exclusively within the hospital at any time.

To measure the effects of *patient letters*, a patient questionnaire was developed. The elements used for this analysis included:

1. Sociodemographic items covering variables such as age, gender, and level of education (see Appendix A). For the analysis, level of education was divided into the categories *low* (no vocational qualification, basic or lower secondary school), *medium* (secondary school, *high* school or apprenticeship) and *high *(university of applied sciences, university or doctorate).
2. The Rasch-scaled short form HLS-EU-Q16 [26] of the European Health Literacy Survey (HLS-EU-Q47) [27]. Following the HLS-EU-Q16 analysis method, the four-level response categories (*very difficult, fairly difficult, fairly easy, very easy*) were dichotomized, so that each of the 16 items obtained a value of 0 or 1. For every participant, all 16 items were added together to form the overall score. A value of 0 corresponds to the worst possible health literacy, whereas a value of 16 corresponds to the best possible. The threshold values for dividing the overall score into *inadequate* (0–8), *problematic* (9–12), and *sufficient* health literacy (13–16) were adopted analogously from Röthlin et al. [26]. All participants were given two further questions to assess the need for a comprehensible explanation of medical reports (1) following an inpatient stay and (2) following an outpatient visit.
3. 12 self-developed questions to evaluate the *patient letter* (for IG participants only; see Appendix B). The aspects “helpful”, “comprehensible” and “informative” were rated on a four-point scale (*fully applies, rather applies, rather does not apply, does not apply at all*).

To explore patients’ attitudes towards *patient letter* and to identify potential needs for improvement, a self-generated guideline for qualitative interviews was developed (see Appendix C).

### Statistical analysis

Descriptive statistics were used to summarize demographic and clinical characteristics. For the analysis of the first hypothesis, we used the nonparametric Mann Whitney *U* test due to its low restrictive assumptions to compare HLS-EU-Q16 scores between IG and CG. To assess group differences in the proportion of participants with *inadequate*, *problematic*, and *sufficient* health literacy levels, Pearson’s chi-squared test was used. For the analysis of the second hypothesis, Pearson’s chi-squared test was performed to examine the association between educational level and the degree of detailed reading. Frequencies and percentages were calculated to evaluate patients’ perceptions of the patient letter in terms of being understandable, helpful and informative. All quantitative analyses were conducted using SPSS, with a significance level set at *p* < .05.

For qualitative analyses, the transcribed interviews were sighted by the interviewer and two additional reviewers. A coding system was developed based on the interview guideline (see Appendix C) and emerging themes, following the content analysis approach by Kuckartz [28]. Using the MAXQDA software [29], key steps included identifying relevant text passages, developing main categories, coding the material, and refining subcategories inductively. The data were then reduced, paraphrased, and interpreted, ensuring reliability through multiple coders.

## Results

### Descriptive statistics

The majority of participating patients was male (61.1%) and over 65 years old (66.8%), which can be considered representative of an average cardiologic patient [30]. 18.4% indicated a low, 55.7% a medium and 22.5% a high level of education. On average, patients were hospitalized for 6.6 days (range: 1–54 days). There were no significant differences between IG and CG in terms of age, gender, level of education, presence of a long-term illness, migration background, health profession, self-assessed health status and number of days in hospital. The detailed sample description is displayed in Table 1.

**Table 1:** Sample description

	IG (*n* = 363)	CG (*n* = 375)
Gender		
Male	217 (59.8%)	234 (62.4%)
Female	126 (34.7%)	122 (32.5%)
N/A	20 (5.5%)	19 (5.1%)
Age		
20–45 years	9 (2.5%)	12 (3.2%)
46–65 years	92 (25.3%)	111 (29.6%)
Over 65 years	253 (69.7%)	240 (64.0%)
N/A	9 (2.5%)	12 (3.2%)
Level of education		
Low	68 (18.7%)	68 (18.1%)
Medium	215 (59.2%)	196 (52.3%)
High	69 (19.0%)	97 (25.9%)
N/A	11 (3.0%)	14 (3.7%)
Long-term illness (over 6 months)	283 (78.0%)	302 (80.5%)
Self-assessed health status		
Good/very good	100 (27.5%)	105 (28.0%)
Medium	193 (53.2%)	194 (51.7%)
Bad/very bad	56 (15.4%)	52 (13.9%)
N/A	14 (3.9%)	24 (6.4%)
Number of days in hospital		
1 day	43 (11.8%)	61 (16.3%)
2–5 days	153 (42.1%)	147 (39.2%)
6–10 days	88 (24.2%)	92 (24.5%)
Over 10 days	79 (21.8%)	75 (20.0%)

*Note: *Numbers in absolute frequencies (relative frequencies in %). IG: intervention group. CG: control group. N/A: no answer

Prior to the study, it was determined that the HLS-EU-Q16 overall score should be calculated only if no more than two items were missing. As 12.5% of the returned HLS-EU-Q16 questionnaires turned out to be incomplete with at least two items missing, no overall score could be calculated for these cases. This resulted in a reduction of sample sizes in IG (*n* = 363 to *n* = 317) and CG (*n* = 375 to *n* = 329) regarding the hypothesis 1.

### Results regarding hypothesis 1

**Fig. 2:**
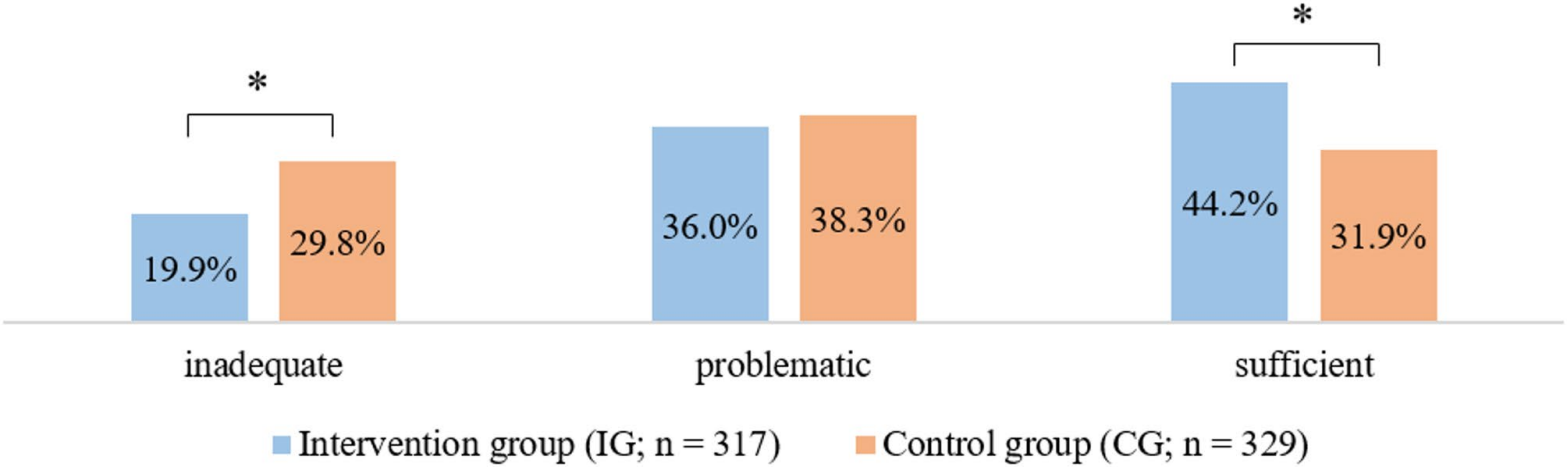
Relative frequencies of health literacy levels (in %) measured with the self-report questionnaire HLS-EU-Q16. *Note*. * indicates a statistically significant effect at *p* < .05

Comparing the mean overall HLS-EU-Q16 score in both groups with the Mann-Whitney *U *test, health literacy was significantly higher in the IG (*M*_IG_ = 11.3, CI = [11.0, 11.7]) than in the CG (*M*_CG_ = 10.5, *CI* = [10.1, 10.9]; *p* = .002). In line with this, IG participants were significantly less likely to have *inadequate *health literacy (19.9% vs. 29.8% in control group, *p* = .002) and significantly more likely to have *sufficient *health literacy (44.2% vs. 31.9% in control group, *p* = .002; see Fig. 2).

### Results regarding hypothesis 2

93% of IG participants reported to have read the *patient letter* in detail, whereas the remaining 7% reported to have skimmed it briefly. A higher educational level was associated with a more detailed reading of the *patient letter* (*p* = .048; Person’s chi-squared test): all patients with a high level of education stated to have read the *patient letter* in detail, compared to 86% with a low level of education. When asked whether the *patient letter* was perceived as helpful, comprehensible and informative, over 90% of answers were either* fully applies or rather applies* (see Table 2). In addition, patients without a long-term illness were more likely to rate the *patient letter* as helpful than those with a long-term illness (100% vs. 92%, *p* = .022).

**Table 2:** Evaluation of patient letters by intervention group participants

	“The *patient letter* was…”		
	… helpful	… comprehensible	… informative
*fully applies*	207 (57.0%)	231 (63.6%)	216 (59.5%)
*rather applies*	115 (31.7%)	98 (27.0%)	99 (27.3%)
*rather does not apply*	20 (5.5%)	15 (4.1%)	23 (6.3%)
*does not apply at all*	3 (0.8%)	1 (0.3%)	2 (0.6%)
N/A	18 (5.0%)	18 (5.0%)	23 (6.3%)
*N*	363	363	363

*Note: *Numbers in absolute frequencies (relative frequencies in %). N/A: no answer

The *patient letter* was frequently used in conversation with third parties: 73% of IG participants stated to have shown or spoken about the *patient letter* to at least one other person. Furthermore, 89% of IG participants found the descriptions of examinations or and 90% the descriptions of diagnoses *very helpful* or *rather helpful*, with 7% vs. 8%, respectively, finding them *rather unhelpful* or *not at all helpful*. 77% of patients rated the scope of the *patient letters* as just right, 13% as too extensive and 10% as too brief. Across both study groups, virtually all participants (over 99%) considered a comprehensive written explanation of medical reports after every inpatient stay to be *important or rather important*. In contrast, only 76% considered it important or rather important after outpatient visits.

Analysis of the qualitative interviews showed that the majority of interviewees (11 out of 15: ID 3, 4, 5, 6, 8, 9, 10, 11, 12, 13, 15) expressed a positive attitude towards the *patient letter*. The most frequently mentioned reasons for this were an improved understanding of one’s own medical history, the clear and understandable writing style for laypeople (numerous mentions), as well as the high suitability for “uninformed”, recently diagnosed and older patients (ID 8, 15).

[…] For me it was a very good addition to the knowledge I had before […]. [I]t was visually nicely done, […] it contained information, also well-explained information, so I was satisfied with it. (male, 50–55 years old, ID 3)

Comprehensibility of the *patient letters* was also assessed during the interviews. Ten interviewees provided information on comprehensibility and all of them reported the *patient letter* to have been comprehensible to them without further help. The letter was reported to be written in an easily understandable manner (ID 2, 9), to explain the illness, procedure or examination well (ID 3, 6, 10) and to be more comprehensible than the standard discharge letter (ID 1, 10, 11). The interviewees were asked whether they had received new information from the *patient letter*. Five interviewees (ID 1, 3, 4, 6, 9) reported that they had experienced an increase in knowledge, whereas four interviewees reported to have not experienced any increase in knowledge, as they were already familiar with the content (ID 3, 8, 11, 15). One interviewee reported that he had already taken the information from the standard discharge letter (ID 8). The interviewees considered the greatest personal gain from the *patient letter* to be a better understanding of the examinations and treatments carried out and their own illness or diagnosis (ID 1, 3, 4, 6, 10, 11).

The explanation of why this [medical procedure] was necessary, why it had to be done, and what I actually have – that was absolutely perfect for me. The whole explanation, really: why, for what reason, and on what grounds. It really gave me a lot, I must say. (female, 70–75 years old, ID 6)

Lastly, potential needs for optimization were identified based on the interviews. With regard to the writing style, overly simplified wording (ID 2, 14) and overly frequent repetition of text modules (ID 3) were criticized. Some interviewees wished for less extensive explanations (ID 3, 8, 12, 15). The desire for more individuality in the text modules was also expressed (ID 14). Other suggestions included an incorporation of more visual illustrations for a better understanding (ID 2, 14) and the listing of contact data in case of questions on side of the patients (ID 2, 4). It was also mentioned that receiving the *patient letter* even earlier after discharge would be an advantage (ID 3). Half of the interviewees had no suggestions for improvement (ID 4, 5, 7, 8, 12, 11, 13).

[…] The very simplified presentation of the condition, broken down to an extremely basic level for comprehension, seemed a bit overdone to me – in terms of understanding. (male, 65–70 years old, ID 14)

## Discussion

### Summary of results

The present study compared cardiologic patients who were given a conventional discharge letter only (CG) with those who additionally received a personalized, automatically generated, easy-to-understand patient letter via post after hospital discharge (IG). The study not only revealed that patients positively value the reception of *patient letters*, but it also demonstrated a measurable association of these letters with enhanced health literacy levels.

Our first hypothesis was that receiving a *patient letter* after an inpatient stay would increase health literacy in patients. Results confirmed the hypothesis, as IG participants had a higher overall score in self-reported health literacy than CG participants. In support of this finding – following Röthlin et al.’s [26] subdivision in *inadequate, problematic* and *sufficient *health literacy – IG participants showed a lower frequency of *inadequate *and a higher frequency of *sufficient* health literacy than CG participants. These results extend findings of the previous studies that have demonstrated positive effects of patient-friendly discharge letters on improving patient compliance [17], level of information and disease-related understanding [18, 19, 21], and sense of security [23]. Together with our study, evidence suggests that the provision of tailored, comprehensible discharge information can play a crucial role in enhancing patients’ understanding of their health and treatment, particularly in the context of chronic conditions like cardiovascular diseases.

Our second hypothesis was that patients would perceive the *patient letters* as helpful, comprehensible and informative. A high number of the participants of all educational levels (86–100%) stated to have read the *patient letter* in detail. The results confirmed the hypothesis 2, as the majority of IG participants (over 90%) responded either *fully applies* or *rather applies* to the question of whether the *patient letter* had been helpful, comprehensible and informative. This impression is supported by the fact that a large majority of IG participants found the descriptions of the examinations (89%) or diagnoses (90%) *very helpful or rather helpful*. The conducted qualitative interviews supported this result, as most interviewees expressed a positive attitude towards the letters and mentioned them to be well comprehensible. Around half of the interviewees that commented on the change in knowledge regarding their health status associated to the *patient letters* reported an increase, while the other half reported no increase. This might be explained by the fact that cardiac health issues usually are of chronic matter and patients are typically informed about their condition for a long period of time. Together, our results show a high satisfaction with patient letters, which is backed up by international literature, according to which patient-friendly discharge letters are well received and can increase patients’ satisfaction [18–20, 22].

While many patients in this study valued the *patient letters* for their clear explanations of diagnoses and procedures, some reported that certain sections contained information they already knew, that was too repetitive or that they found overly simplified. This suggests that while the letters successfully enhance understanding for many, their effectiveness may vary depending on prior knowledge and individual informational needs. The fact that a majority of IG participants (73%) had mentioned the *patient letter* in conversation with third parties emphasizes the potential of these letters to promote verbal exchange about personal health status and thus support patients and their peers in health communication. Since virtually all participants independent of their study group expressed a desire for a clear explanation of medical reports after an inpatient stay, an overall high need for comprehensible health information on part of the patients is indicated. Overall, these results emphasize the importance of easy-to-understand patient information after discharge that meets different patient needs and preferences. While some aspects, such as overly simplified wording, were identified as areas for improvement, *patient letters* demonstrate their potential to fulfill these different requirements, with further refinement possibly further enhancing their effectiveness.

### Limitations and strengths

This study has several limitations and strengths. The cross-sectional design of this study results in certain limitation for interpretation: As health literacy was assessed at a single time point after post-intervention, we cannot draw definitive conclusions about the causal effects of the intervention due to the lack of evidence for change over time. Further, we relied on a self-reported health literacy measure, which introduces potential bias. The HLS-EU-Q16 is a well-established tool for measuring self-reported general health literacy, providing valuable insights into individuals’ overall ability to understand health-related information. However, there is a growing demand of disease-specific health literacy instruments, as they could more accurately capture the specific challenges patients face in managing their health conditions. Such tools might be better equipped to evaluate patients’ understanding of information tailored to their particular disease-specific needs, improving the relevance and effectiveness of interventions. Therefore, while the HLS-EU-Q16 used in this study serves as a broad measure, it may not fully address the complexities of health literacy in specific patient groups.

Since one eighth of the returned HLS-EU-Q16 questionnaires were incomplete (> 2 missing items) and the overall scores could not be calculated for those responses, the previously determined sample size for the hypothesis 1 was not met (*n* = 316 and *n* = 329 instead of calculated *n* = 357 each). A post-hoc power analysis was conducted to assess the statistical power with this reduced sample. Results showed that due to the larger-than-estimated effect between IG and CG (Cohen’s *d* = 0.23) regarding HLS-EU-Q16 scores, a satisfactory post-hoc power of 84% could be reached.

A potential limitation to consider is that all participants had actively decided upon admission to receive additional health information after discharge, suggesting a selection bias which could lead to an over-representation of positive evaluations. Thus, future studies are needed to assess whether the positive reception observed in this study is generalizable to all patients, independent of their health status and initial attitude towards *patient letters*.

A noteworthy strength of the *patient letters* used in this study is their automated production by “Was hab’ ich?”. This practice stands in contrast to the previous study [19], in which medical staff compiled the letters manually and the time required per letter was approximately 30 to 120 min. Based on the method of automated generation of the *patient letters*, this effort could be eliminated in the current study. This successfully paves the way for the introduction of *patient letters* into standard care. Current artificial intelligence (AI) based methods enable patients to translate their conventional discharge letters into layperson friendly text. In contrast, the *patient letters* examined in this study do not provide a mere translation of discharge letters, but deliver reliable and medically substantiated explanations based on discharge data. The content blocks are written by a medical editorial team and undergo a quality assurance process with double-checking, making them more trustworthy than explanations generated by AI systems that may not always provide accurate information. Thus, the rule-based approach used for these *patient letters* allows for the generation of reliable information without the need for checking each individual document, which would be necessary when using an AI system. This aspect is a key benefit of *patient letters* compared to AI systems.

Multiple other studies [10, 12, 13, 27] have internationally shown limited health literacy to be associated with poorer health outcomes and higher healthcare expenditure. So even though the cost-benefit ratio of *patient letters* was not investigated in this study, their time and cost-efficient production suggests that an introduction into standard care could result in savings for the healthcare system. Increasing health literacy in the public is a declared goal of the *National Action Plan on Health Literacy for Germany*. In the course of this declaration, the involved experts called for (intervention-based) research on the complex, multidimensional and relational construct of health literacy to be intensified [16, 31]. In this regard, it is noticeable that this study is one of only a few intervention studies to have measured effects on health literacy as an outcome variable. The study showed that *patient letters* are a promising approach for improving health literacy of patients.

### Conclusion and outlook

This randomized controlled study with a mixed-methods approach shows that receiving easy-to-understand discharge letters after hospitalization is associated with higher post-intervention health literacy in cardiologic patients. Participants perceived *patient letters* as helpful, comprehensible, and informative. Moreover, the process of producing and distributing the letters was successful, indicating the potential for broader adoption in routine hospital care. In line, the *Innovation Fund of the Federal Joint Committee* has recommended the introduction of *patient letters* into standard care in Germany [32]. Future research should focus on the comprehensive integration of patient letters into existing hospital information systems to increase accessibility across different patient groups and healthcare settings [33]. The introduction of personalized, automatically generated *patient letters* is a promising approach to enhance patient-centered communication and empower patients to better understand health information and navigate the healthcare system.

## Supplementary Information

Below is the link to the electronic supplementary material.

Supplementary Material 1

## Data Availability

The following study materials are available in German and can be obtained from the authors on request: questionnaires for intervention & control group, interview guideline, and code book. An exemplary patient letter can be retrieved online: (https://patientenbriefe.de/downloads). The datasets generated and analyzed during the current study are not publicly available due to the regulations of the responsible data protection authority to ensure the confidentiality of sensitive patient information. Specific subsets are available from the corresponding author on reasonable request.
