# Supplementary material for: Evaluating the impact of easy-to-understand patient letters after discharge on patients’ health literacy: a randomized controlled study
Source: BMC Health Serv Res. 2025 Oct 15;25:1366. doi: 10.1186/s12913-025-13464-4 (PMC12522859; doi:10.1186/s12913-025-13464-4)
Supplement: Supplementary file 1 — Supplementary Material 1 [file 12913_2025_13464_MOESM1_ESM.docx]

**Appendix A: Sociodemographic Questions**

(translated into English)

Please indicate your gender:

□ male

□ female

□ diverse

Please indicate your age:

_________

Are you or your parents born in a country other than Germany/former German territory?

□ yes

□ no

What is your highest level of education?

□ no vocational qualification

□ basic or lower secondary school

□ secondary school

□ high school

□ apprenticeship

□ university of applied sciences

□ university

□ doctorate

Do you work in the field of healthcare?

□ yes

□ no

How often have you spent at least one night in hospital in the last few years? The current stay at the Heart Center Dresden counts.

__________

How would you describe your current health status?

□ very good

□ good

□ medium

□ bad

□ very bad

Do you have a long-term illness or a long-term health problem? The term “long-term” refers to problems that have lasted or could last for 6 months or longer.

□ yes

□ no

**Appendix B: Evaluation of the *Patient Letter***

(translated into English)

You recently received a *patient letter* by post in addition to your discharge letter. The following questions relate to this *patient letter*.

Have you read the *patient letter*?

□ yes, read in detail

□ yes, briefly skimmed

□ no

Has anyone - apart from you - read the *patient letter*?

□ no, nobody

□ yes, one other person

□ yes, two other people

□ yes, three or more other people

□ I don’t know

If yes: Have you spoken to one or more of these people about the *patient letter*?

□ yes

□ no

The *patient letter* was helpful.

□ fully applies

□ rather applies

□ rather does not apply

□ does not apply at all

The *patient letter* was understandable.

□ fully applies

□ rather applies

□ rather does not apply

□ does not apply at all

The *patient letter* was informative.

□ fully applies

□ rather applies

□ rather does not apply

□ does not apply at all

My expectations regarding the *patient letter*:

□ were exceeded

□ were met

□ were partially met

□ were not met

□ I had no expectations

□ I don’t know

Scope: I found the *patient letter*...

□ far too extensive

□ a little too extensive

□ just right

□ a little too brief

□ far too brief

I found the description of the examinations carried out in the *patient letter*...

□ very helpful

□ rather helpful

□ rather unhelpful

□ not at all helpful

□ I don’t know

I found the description of the diagnoses in the *patient letter*...

□ very helpful

□ rather helpful

□ rather unhelpful

□ not at all helpful

□ I don’t know

I find being able to take home a comprehensible, written explanation of the medical reports after every visit to the doctor...

□ important

□ rather important

□ rather not important

□ not important

□ I don’t know

I find being able to take home a comprehensible, written explanation of the medical reports after every hospital stay...

□ important

□ rather important

□ rather not important

□ not important

□ I don’t know

**Appendix C: Interview Guideline**

(translated into English)

**Research Topics:**

A) Perception of hospital stay and discharge

B) Perception of doctor-patient communication and general health information acquisition

C) Evaluation of the *patient letter*

**Table 1: Preparation Using Quantitative Data**

| **No.** |  | **Response** | **Fulfilled** |
| --- | --- | --- | --- |
| 1 | Was a discharge discussion with a physician conducted during your hospital discharge? | No / I don't know | □ |
| 2 | The *patient letter* was... helpful, understandable, informative | Rather does not apply / Does not apply at all | □ |
| 3 | Scope: I found the *patient letter*... | Far too brief | □ |
| 4 | Scope: I found the *patient letter*... | Far too extensive | □ |
| 5 | I found the description of the examinations carried out in the *patient letter*... | Not at all helpful / I don’t know | □ |
| 6 | I found the description of the diagnoses in the *patient letter*... | Not at all helpful / I don’t know | □ |

**Introduction:**

- Thank the participant for joining the study and taking the time for the interview.
- Introduce yourself as a research associate from the Department of General Practice at TU Dresden.
- Explain the study’s aim to assess the impact of the *patient letter* on patients.
- State that the study is publicly funded.

Context:

- Remind the participant that he/she was discharged from the cardiology clinic about [X] weeks ago and received a *patient letter* from the research team.
- Indicate the intention to discuss their experiences during the hospital stay and their thoughts on the *patient letter*.

Confidentiality:

- Ensure the participant that all responses will remain confidential and will be documented in a way that ensures anonymity.
- Emphasize that there are no right or wrong answers, and their personal opinion is crucial to the study.

Interview Logistics:

- Inform the participant that the interview will last approximately 20-30 minutes.
- Request consent to record the interview digitally to minimize information loss. If the participant does not agree, note that the conversation will be documented manually.

Recording Consent:

- Confirm that the participant is comfortable with the recording and ask if they have any questions before starting.
- Tell the participant that the recording starts now.

[Start the recording]

**Interview: Guiding Questions, Prompts & Follow-Up-Questions**

A) Perception of hospital stay and discharge

- “You were **admitted to the hospital** in Month [X]. How was your stay?”
- “Did you receive all the important **information about your stay** while you were in the hospital?”
  - “Did you receive information about what to do after discharge?”
- (If a discharge discussion was conducted): “You mentioned that you had a **discharge discussion** with a physician at the end of your hospital stay. Did you feel sufficiently informed during this discussion?”
  - “How did you feel about the discharge discussion at the end of your hospital stay?”
  - “Were you able to ask many questions during this discussion?”
    - „If not, why?“
  - “Were there any specific topics or information you felt were missing?”
    - “If so, what were they?”
- (If no discharge discussion was conducted): “You mentioned that **no discharge discussion** was held when you left the hospital. Did you still receive all the important information you needed?”

B) Perception of doctor-patient communication and general health information acquisition

- „Doctors and patients often speak different ‘languages’ (**doctor-patient communication**). How do you perceive or experience this in your interactions?”
- “How clear or understandable is the **information** you receive **from your doctor**?”
  - “Do you also ask questions? If not, why not?”
  - “What would make it easier for you to ask questions?”
  - “What would make the doctor‘s information easier to understand?”
  - “What would help you better remember or retain the information provided?”
  - “At home, can you still remember all the important information from the doctor?”
- “How easy or difficult is it for you to **find** trustworthy health information?”
  - (Consider access and the reliability of information)
  - “Do you search independently, or do you require help?”
- “Where do you look for health-related information (e.g., diseases, treatments)?”
- “How trustworthy do you find the following sources:”
  - „Media (internet, newspapers, television)?“
  - „Friends or family?“
  - “Medical professionals (general practitioners, pharmacists, nurses)?”
  - „Scientists or experts?“

C) Evaluation of the *patient letter*

- “We sent you our ***patient letter***. What are your thoughts on it?”
  - “What was your **initial reaction and thought** upon receiving the *patient letter*?”
    - “Did you feel surprised, happy, overwhelmed, or anxious? Why?“
- “How do you cope with the information in the *patient letter* today?”
- “What did you find **most important** in the *patient letter*?”
- “Did it contain any new information for you?”
  - “In which areas?”
  - “Were there any surprises?”
- “Was the patient letter understandable and helpful without additional assistance?” (If not, why?)
  - Consider aspects such as readability, use of technical terms, scope, or content.
- “Is there anything you **wish** had been included in the *patient letter*?”
  - “Were there any unclear, missing, insufficient, or excessive details?”
  - “Was the scope or number of pages appropriate?”
  - “Individual information?”
- Follow-Up Questions - if not yet discussed, ask:
  - “You mentioned in the written survey that the *patient letter*’s scope was either ‘too brief’ or ‘too extensive’. What would you want to change about it?”
  - “You indicated in the survey that the *patient letter* was not helpful, understandable, or informative. What do you feel was missing?”
  - “You noted that the description of your examinations or your illnesses in the patient letter was not helpful. What did you miss?”
- “Did you discuss the *patient letter* with anyone else?” (**social impact**)
  - Did you talk about it with your **doctor**?
    - “If so, what was discussed, and why?”
    - “What was the doctor's reaction?”
    - “Did the *patient letter* influence your communication with the doctor? If yes, how?”
  - “Did you talk about it with **friends or family**?”
    - “If so, what was discussed, and what were their reactions?”
- “What do you think is the **main advantage** of the *patient letter* for you **personally**?”
  - “Can you explain why?”
- “What about the patient letter might **bother or annoy** you?”
  - “Can you explain why?”
- “Would you be **interested** in receiving a similar *patient letter* after other medical visits, for example, at your general practitioner’s office?”
  - Why or why not?
- “In what **format** would you prefer the *patient letter*?”
  - Paper version?
  - Digital format (e.g., for phone or tablet)?

**Conclusion of the Interview:**

- “We have now reached the end of the interview. Is there anything else you would like to add or share?”
- “Thank you very much for taking the time to participate in this interview!”

[End the recording]
